# Gonorrhœa—Its Bathology and Treatment

**Published:** 1878-05-20

**Authors:** C. C. Godshaw


### GONORRHEA—ITS PATHOLOGY AND TREATMENT.

BY C. C. GODSHAW, A.B., M.D.

(Read before the Louisville Academy of Medicine, April 19, 1878.)

---

To the young practitioner especially is the study and proper treatment of gonorrhea deserving of consideration, as it is to him that patients usually come for relief. Almost every doctor claims to possess a sovereign cure, and yet we not unfrequently find cases that have proven rebellious to all treatment, and the patient, disappointed and disgusted, lets the disease take its own course.

I propose this evening to show as brifly as possible that success depends less on the medicinal agents given than on close attention to cleanliness and the thoroughness of application of the means employed.

PATHOLOGY.

Gonorrhea in the male is an inflammation of the mucous membrane lining the urethra analogous to catarrhal inflammation which invades the conjunctiva. It is a local inflammatory trouble requiring local treatment for its cure. It is virtually an urethritis, but not always of a specific nature. Although writers on the etiology of this disease admit its infectious and contagious nature, yet its specialty in all cases is to be doubted. In proof of this I could report many cases whose history and origin I have traced, and know have not been due to the specific virus. As it is a well-known fact that almost all women, be they chaste or unchaste, have leucorrhea, and since as yet the microscope has failed to reveal any difference between gonorrhea and simple urethritis, the history alone can determine the nature of the cases. Gonorrhea should therefore be classed as an urethritis—a tumified and vascular condition of the lining of the urethra discharging a muco-purulent secretion.

TREATMENT.

Believing as I do in the local nature of the diease, I do not recommend the anti-blenorrhagic treatment, as few will tolerate it, and besides with me it has not worked any good results. The so-called abortive treatment—caustic injections and applications—should also be discountenanced, as they do no little harm.

Gonorrhea, like other catarrhal inflammations—as, for instance, ordinary conjunctivitis—can be cured with mild, weak, and soothing astringent injections properly used, and with due attention to hygiene and cleanliness. Certainly when indicated in constitutional derangements—as malaria, anæmia, rheumatism, etc.—tonics and appropriate medication are necessary. It should also be seen that the bowels are kept soluble and kidneys act freely. The following is a favorite combination with me, and cures, when used as directed, in from ten days to three weeks, often sooner:

℞ Sulph. zinc........ } aa grs. ij-iij;

Tannic acid........ }

Sulph. morph........ grs. iij-vi;

Sulph. atrop.......... gr. ss-j;

Distilled water....... oz. vj.

Dissolve. Direct to inject every two hours.

Since I have used the atropia in combination with morphia, in weak astringent injections, I find, through its valuable anodyne and antispasmodic properties, that the tendency to chordee is controlled, while the pain produced by the passage of urine is considerably mitigated.

If there is excessive pain during micturition, the following formula, much used by Zeissel and Sigmund, of Vienna, may be prescribed:

℞ Ext. cannabis Indic. } aa grs. iv;

Ext. hyoscy. sem..... }

Sacch. alb............ oz. ss.

M. Ft. chart No. 9. Sig. One every three hours.

In using the injection the patient should be shown very minutely the method of using the syringe. The doctor should always inject for him the first time, and afterwards if necessary, thus seeing that he performs it properly, and as the symptoms and discharge decrease so should the number and frequency of the injections. After using the syringe, so plainly described by Bumstead, the instrument should be withdrawn, and the medicated liquid allowed to remain for a few minutes.

Before doing this, however, one rule must be implicitly followed—to urinate before injecting. It is also a good plan to inject simple water just previous to using medicated fluid, thus insuring thorough cleansing of canal.

In regard to syringes, the hard rubber is preferable. Its nozzle should be about one half inch in length and smooth, so as not to lacerate the already diseased tissue. The patient should also be advised to wear a suspensory bandage during the treatment. If the bandage can not be conveniently had or bought a very ingenious substitute is found in the shape of the heel of a cotton sock, to which are attached four cords or pieces of tape.

### HYGIENE AND CLEANLINESS.

In a person whose glans penis is covered with a long prepuce too much attentien to thorough cleanliness can not be exercised. Frequent ablutions must be practiced. In such a case the cure is undoubtedly retarded by the meatus urinarius being occluded by the necessarily inflamed preputium and the constant irritation of the accumulating discharge and decomposed secretion. Such disgusting signs are seen on persons who otherwise are scrupulously clean! I find that the circumcised, or even those whose glans are partially covered, yield more readily to treatment. The sanitary influence of circumcision in the treatment of gonorrhea alone, in my observation, is a great factor.

### DIET AND HABITS.

Moderation and temperance should be advised. Wholesome and nutritious food must be taken, whilst the idea of starving out the disease is simply ridiculous. If the patient be accustomed to liquors, malt or otherwise, or tobacco, I find by depriving him entirely of these the disease is retarded in its cure. If when used in excess with a debilitating effect, certainly they must be interdicted, as well as society and thoughts that tend to excite the sexual organs.

It is also well to observe, as the majority of cases are in persons who, from business or other relations, can not lie down, we must enjoin as much rest as possible during treatment, and also prohibit sexual intercourse until several weeks after the cessation of the discharge.

In conclusion, I would add that we cannot be too minute and clear in our instructions, for just in proportion to these directions will the intensity and duration of his case be lessened.—*Lou. Med. News.*

